# Editorial: Genomic and biotechnological interventions for the concurrent improvement of stress resilience and seed-associated traits in crops

**DOI:** 10.3389/fpls.2023.1359918

**Published:** 2024-01-08

**Authors:** Harmeet Kaur, Rajeev Ranjan, Pallavi Singh, Prafull Salvi

**Affiliations:** ^1^National Institute for Plant Biotechnology, Indian Council of Agricultural Research (ICAR), New Delhi, India; ^2^Department of Horticulture and Landscape Architecture, Purdue University, West Lafayette, IN, United States; ^3^School of Life Sciences, University of Essex, Colchester, United Kingdom; ^4^Agriculture Biotechnology Department, National Agri-Food Biotechnology Institute (NABI), Punjab, India

**Keywords:** abiotic and biotic stresses, CRISPR (clustered regularly interspaced short palindromic repeat)/Cas9 (CRISPR associated protein 9)-mediated genome editing, multi-locus genome-wide association studies, ROS - reactive oxygen species, seed & seedling, phytohormonal balance

Navigating the challenges posed by extreme climate variations and the rising global population is paramount in this century. To ensure food security for the rising global population in context of depleting resources and unfavourable environmental conditions is a challenging feat. Moreover, plant growth is shaped by a complex interplay of biotic and abiotic factors, encountered by a plant during reproductive stages, profoundly impacting seed yield and quality. This, in turn, resonates through agricultural productivity, leading to suboptimal land utilization and resource allocation. In response to these imminent challenges, researchers across the globe have engaged in the quest for the generation of climate-resilient crops. Their efforts are focussed on advancements in increasing photosynthesis efficiency, enhancing plant biomass, increasing seed yield, elevating nutritional attributes, and grain quality, utilizing both traditional breeding and cutting-edge molecular genetics approaches (Manna et al., 2021; Salvi et al., 2022b). Besides, the advent of gene-editing technologies has further reinforced these endeavours, showing promising outcomes. The cellular machinery modulates different aspects of plant metabolism, including sugar signalling, phytohormone regulation, transcription factors, ROS metabolism, etc, to adapt the stressful conditions and tackle yield loss (Kaur et al., 2021; Salvi et al., 2021; Salvi et al., 2022a; Kajal et al., 2023). This Research Topic determines the convergence of two pivotal facets of crop protection programs— strengthening environmental resilience and improving seed-associated traits. The Research Topic received six original research and one review article that focus on important aspects to amalgamate diverse studies that underscore the interconnection of these research themes.

Reactive Oxygen Species (ROS) play a crucial role in plant responses to pathogen attacks, with variations in patterns for necrotrophs and biotrophs. While ROS, in regulated concentrations, serve as signalling molecules, excess levels of ROS is detrimental, affecting cell structure and membrane stability thus lowering plant yields. Plants have intrinsic mechanisms to counteract excess ROS and maintain homeostasis. Microbes, especially endophytes, contribute to ROS homeostasis under biotic stress through various mechanisms, producing antioxidants and inducing host plant machinery for ROS scavenging. An excellent literature review by Sahu et al. successfully underlines the potential of using endophytes as inoculants for countering plant stress and thus improve yields. They discuss the recent developments in ROS homeostasis using endophytes while uncovering the intricate cross talk between ROS production, signalling and host-plant responses to oxidative stress during biotic stress. Another study described the role of rhizospheric and endophytic bacteria in mitigating salinity stress compromised germination in rice (Gupta et al.). The group reported the isolation and characterization of twenty-seven bacterial cultures for their ability to alleviate salinity stress. *Brevibacterium frigorito*lerans, W19 and *Bacillus safensis*, BTL5, added with melatonin, improved seed germination and seedling vigor under salinity stress with enhanced chlorophyll, proline, enzyme levels and better plant growth parameters. They also conducted gene expression studies to reveal the modulated expression of key genes responsible for improving these traits. The study provides interesting insights into the use of combination of microbes aiding in plant growth and melatonin which could provide additive effect thus offering better potential for developing stress-alleviating bioformulations.

Heat stress is another important factor contributing to massive yield losses in crop plants. Heat Shock Factor Binding Proteins (HSBPs) are extensively known for their role during heat stress responses. However, Muthusamy et al. explored the role of *Brassica rapa* heat shock factor binding protein 1 (BrHSBP1) in controlling both seed and pod development. Various abiotic stresses and phytohormones differentially regulate *BrHSBP1* expression. The study looked at the overexpression of *BrHSBP1* which enhanced pod and seed size, while -Cas9 edited knock-out lines showed aborted seed and pod development. The in-depth investigation of the transcriptome and protein-protein interaction revealed differential expression of genes and interaction with proteins related to plant reproductive structure development thus, reinforcing the involvement of *BrHSBP1* in seed development in *Brassica rapa* which demonstrates huge potential for concomitant gains in yield and stress tolerance in this economically important crop.

Biotic stresses have always been a major contributor to crop losses and breeding between resistant and susceptible varieties provides a means to transfer the QTL/Loci responsible for resistance among these varieties. Sinha et al. developed a recombinant inbred line (RIL) resistant against bacterial blight disease of rice caused by *Xanthomonas oryzae* pv. oryzae (Xoo), which is a critical factor for yield loss in rice. Quantitative Trait Loci (QTL) mapping utilising this RIL mapping population developed from crosses of Samba Mahsuri (susceptible) and IR 75084-15-3-B-B (resistant), identified a major locus Xa48t on chromosome 11L associated with the resistance. They reported Os11g0687900, an NB-ARC domain-containing protein-encoding gene as a promising candidate for the resistance phenotype. The authors also developed a co-segregating PCR-based INDEL marker, Marker_Xa48 that can be used for marker-assisted breeding for Xa48t thus offering insights into the genetic basis of resistance along with a practical marker for breeding resistant rice varieties.

Multi-Locus Genome-Wide Association Study (ML-GWAS) is a valuable tool for dissecting the genetic basis of complex seed-associated traits. Flaxseed/linseed *(Linum usitatissimum*) is an important oilseed crop and improvement in its seed weight traits have obvious positive economic implications. Saroha et al. utilized ML-GWAS to further our understanding of the genetic architecture underlying the TSW trait in linseed. The group identified Quantitative Trait Nucleotides (QTNs) associated with Thousand-Seed Weight (TSW) in flaxseed/linseed. A study that involved rigorous field evaluations across five environments over multiple years conducted on a panel of 131 accessions, identified 84 unique significant QTNs for TSW, with 30 stable QTNs explaining up to 38.65% trait variation. Among the twenty-three candidate genes identified for TSW were transcription factors, kinases, and ubiquitin transferases. This paves the way for precision breeding by facilitating the selection of plants with desired seed traits in breeding programs.

Functional characterization of gene families allows an overall understanding of their functions under different physiological conditions which provide the valuable information on the potential candidate genes for developing stress tolerant crops. Two such studies by Ambadas et al. and Manna et al. explored the functional diversity of AHL (AT-hook motif nuclear localized) genes and PIN protein genes respectively. Ambadas et al. identified twenty AHL genes in rice and dissected their gene structure, evolution and transcriptomic analysis in various tissues and developmental stages in rice. OsAHL protein interactions co-regulate vital processes, including flowering, reproductive organ development, and photosynthesis. OsAHL genes are also shown as upregulated under drought and salt stress, particularly in the NL44 genotype. Interestingly, Manna et al. explored the role of PIN family of proteins during drought and salinity stress in rice. Traditionally, PIN proteins are known for auxin transport in plants and regulate key developmental processes in plants thus playing a pivotal role in shaping plant architecture. Genome-wide analysis revealed twelve *PIN* genes distributed across eight chromosomes and their involvement in auxin-dependent modification of root architecture. PIN5C and PIN9 were upregulated during salt and drought stress and could be common targets for both stresses. These insights into the abiotic stress regulation of PIN proteins will pave the way for improving stress response in rice with desired root architecture.

Overall, this Research Topic represents the collection of studies that highlight the intricate interplay between environmental adaptability in terms of both abiotic and biotic stressors and the genetic underpinnings governing seed-associated traits, advancing towards sustainable food security in the face of a changing climate.

## Author contributions

HK: Writing – review & editing. RR: Writing – review & editing. PSi: Writing – review & editing. PSa: Writing – original draft, Writing – review & editing.
